# Unilateral Adult Xanthogranulomatous Infiltration of the Conjunctiva, Limbus and Sclera Leading to a Combined Ophthalmic Artery and Central Retinal Vein Occlusion

**DOI:** 10.2174/1874364101711010362

**Published:** 2017-11-23

**Authors:** Burak Unlu, Ziya Ayhan, Banu Lebe, Suleyman Men, Ismet Durak, Ali Osman Saatci

**Affiliations:** 1 Department of Ophthalmology, Dokuz Eylul University, İzmir, Turkey; 2 Department of Pathology, Dokuz Eylul University, İzmir, Turkey; 3 Department of Radiology, Dokuz Eylul University, İzmir, Turkey

**Keywords:** Conjunctiva, Exudative retinal detachment, Limbus, Retinal artery occlusion, Retinal vein occlusion, Sclera, Xanthogranulomatous infiltration

## Abstract

**Objective:**

To describe the features of a female patient with a biopsy-proven xanthogranulomatous infiltration of the conjunctiva, limbus and sclera who had an exudative retinal detachment, combined ophthalmic artery and central retinal vein occlusion unilaterally.

**Method:**

A-53-year old otherwise healthy woman presenting with a painful visual loss in her right eye underwent an ophthalmic examination, meticulous systemic work-up and histopathologic assessment.

**Results:**

Ophthalmic examination revealed multiple subconjunctival masses, upper limbal infiltrations, trace cells in the anterior chamber, pale looking posterior fundus, 360 degree scattered retinal hemorrhages and marked exudative retinal detachment in her right eye. Left eye was completely normal.A biopsy taken from one of the subconjunctival masses demonstrated a diffuse infiltration of the histiocytes and this was interpreted as a xanthogranulomatous infiltration with the help of immunohistochemical staining techniques.

**Conclusion:**

Present case is the only reported adult case with xanthogranulomatous-like infiltration of the eyeball featuring both anterior and posterior segment involvement without any concomitant major systemic disturbances.

## 1 INTRODUCTION

Histiocytic disorders can be classified into three categories: Class I (Langerhans cell histiocytosis-histiocytosis X spectrum), Class II (Histiocytosis of mononuclear phagocytes other than Langerhans cells) and Class III (Malignant histiocytic disorders) [1]. Adult xanthogranulomatous ocular disease is a class II type disease [2]. It is a very uncommon disorder and generally affects the middle-aged individuals without any sex predilection. It can present as subcutaneous, subconjunctival and periocular xanthochromic infiltrates with varying sized mass formation.

We hereby report a 53-year-old woman who had unilateral biopsy-proven adult xanthogranulomatous infiltration including conjunctiva, limbus and sclera resulting in a combined ophthalmic artery occlusion and central retinal vein occlusion.

## 2 REPORT OF A CASE

A 53-year-old otherwise a healthy woman was referred to us with a severe visual loss of a week duration associated with a month-long, red eye, lid puffiness and painful eye movements in her right eye. Her past and family history was unremarkable.

**Fig. (1):**
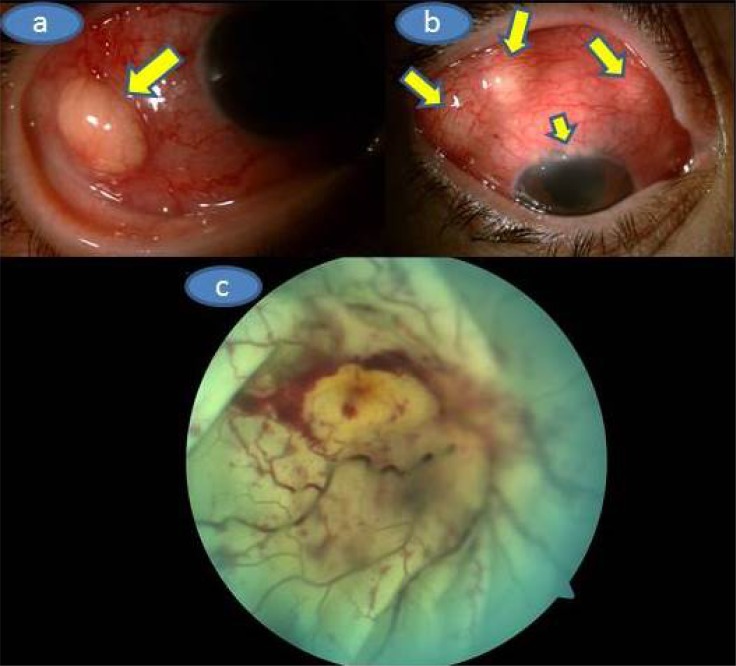
**a)** Color picture of the temporal aspect of the right eye showing the significant hyperemia and whitish-yellowish subconjunctival mass (arrow), **b)** Multiple subconjuctival whitish yellowish masses and upper limbal multiple infiltrations (arrows), **c)** Color fundus picture of the right fundus depicting the massive exudative retinal detachment, palish optic disc with fuzzy borders, 360 degrees scattered retinal hemorrhages and whitish looking entire retina.

**Fig. (2):**
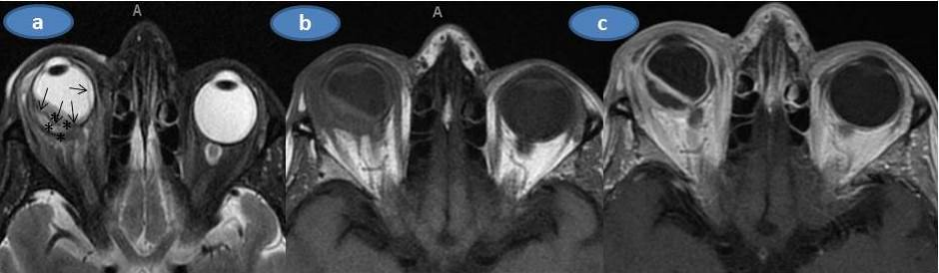
Orbital MR exam; **a)** The axial T2 weighted image; The right globe is deformed and the sclera is thickened especially in the posterior lateral aspect. The right retina looks thickened, undulating and detached (arrows) that is more prominent on the temporal side (arrows). The low T2 signal (relative to the vitreous) material (marked with asterix) has been deposited in both inside and outside of the detached retina implying the infiltrative nature of the deposition. Retrobulbar fat tissue, extraocular muscles and the optic nerve look unaffected. The eyelids on the right side look mildly edematous (thickened with a high signal), **b)** Axial T1 weighted image replicates the above-mentioned findings albeit less clearly. The subretinal collection has an intermediate signal intensity similar to the extraocular muscles, **c)** Axial postgadolinium T1 weighted image shows the thickening and contrast enhancement of the sclera and the retina while the subretinal collection did not exhibit enhancement.

On our examination, Herthel exophthalmometer reading was 19 mm in OD and 14 mm in OS. Upgaze and lateral gaze were minimally restricted in OD. Visual acuity was barely light perception in OD and 8/10 in OS. There was multiple subconjunctival yellowish masses, upper limbal white-yellowish infiltrations, marked ciliary injection, trace cells in the anterior chamber with a clear vitreous in OD (Figs. **1a** and **b**) whereas the left eye was normal upon the slit-lamp examination. Applanation intraocular pressure was 10 mm Hg in OD and 15 mm Hg in OS. Fundoscopic examination showed palish posterior fundus, 360° scattered intraretinal hemorhages and marked exudative detachment in OD while the left fundus was normal (Fig. **1c**). Complete physical examination was normal.Mild neutrophil leucocytosis in which the total leucocyte count was17.2x10^9^/L and mild anemia (Hemoglobin 10.4g/dl, Hematocrit 34.1%) were present. Sedimentation rate was 110 mm/hour. Urine analysis,liver and renal function tests were normal. Chest X-ray was unremarkable.Further work-up was negative for antinuclear antibody, cANCA, PR3ANCA, MPO ANCA, Anti nRNP/Sm, Anti SM, Anti SS-A, Anti SS-b,Anti Scl 70, Anti CENP-B, antinucleosom, antihiston, anti Jo1, antiribosomal P. Serologic tests for syphilis, toxoplasmosis, brucella, rubella, CMV and human immunudeficiency virus were also negative. Skin tuberculin test was 6 mm. Chest and abdominal computed tomography, cranial MRİ and MRİ angiography were normal. Orbital MRI (Figs. **2a**, **b** and **c**) showed that right globe was deformed with significant scleral thickening. There was retinal detachment associated with subretinal deposition of low signal material. This deposition was penetrating into the inner border of the detached retina and the retina looked thickened and had an undulating course. The right eyewall demonstrated a strong contrast enhancement . However, the deposited material did not show contrast enhancement.

**Fig. (3):**
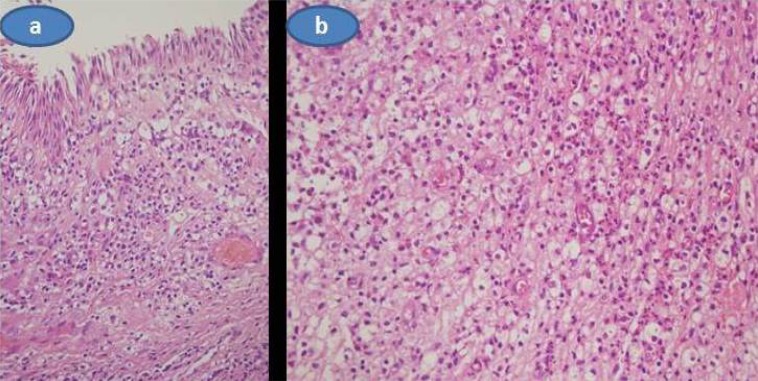
**a)** A diffuse infiltrate of histiocytes with foamy or granular cytoplasm extending towards the lamina propria of the conjunctiva.Original magnificationX40, H and E stain. **b)** A few neutrophils, lymphocytes and plasma were also seen with diffuse infiltrates of histiocytes. Original magnificationX40, H and E stain.Three months after the initial presentation.

A biopsy was performed from one of the subconjunctival masses under topical anesthesia in the operating room. Histopathologic analysis demonstrated that diffuse infiltrate of histiocytes with foamy or granular cytoplasm extending towards the lamina propria of the conjunctiva were present. There was only a few Touton giant cells. (Figs. **3a** and **b**) A few neutrophils, lymphocytes and plasma cells were also seen within the histiocytic infiltrate. Necrosis, lymphoid aggregates, fibrosclerosis, and cholesterol clefts were not seen. Significant mitotic activity was not seen. Immunohistochemical staining revealed the expression of CD68 in the histiocytes while CD1a, pan keratin, actin, and S100 protein were negative. With the help of histopathological evaluation, we reached the diagnosis of unilateral adult xanthogranulamotous-like involvement of the conjunctiva, limbus and scleral tissue that resulted in a combined ophthalmic artery and central retinal vein occlusion.

**Fig. (4):**
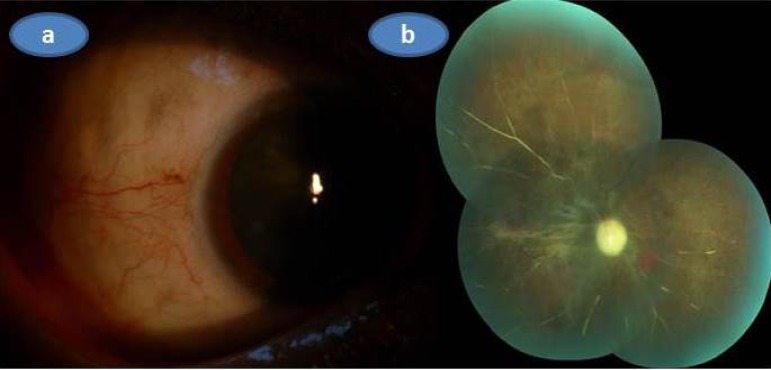
Three months after the initial presentation; **a**) Color picture of the right eye showing the temporal aspect of the right eye that was almost totally quitened, **b)** Composite color fundus picture of the right eye demonstrating the optic atrophy, ghost vessels and some retinal pigment epithelial changes related to severe infarct.

As the visual acuity of the involved eye was only a weak light perception and there was no systemic involvement we put the patient only on hourly topical prednisolone acetate drops with cyclopentolate drop 1% tid without administering any systemic treatment. A week later, the appearance of the anterior segment was markedly improved and topical steroid was given six more weeks with a slow taper. Three months later anterior segment was totally quiet (Fig. **4a**) and there was marked optic atrophy associated with the sequale of the retinochoroidal infarction in the right eye (Fig. **4b**).

## 3 DISCUSSION

Xanthogranulomatous lesions represent a nonneoplastic, reactive proliferation of the histiocytes [3]. There was only a few reports of adult xanthogranulomatous involvement occurred either in conjunctiva or cornea solely [3-12]. However, posterior segment involvement was only reported in two previous papers with the juvenile xanthogranulomatous type of the disorder [13, 14]. Wertz *et al.* [13] described a 20-month-old infant having a blind eye with neovascular glaucoma. Enucleation was performed with the presumption of optic nerve neoplasm. However, microscopic examination revealed an enlarged optic nerve with infiltration of histiocytic cells including Touton giant cells containing large amounts of neutral fat. Occlusion of the central retinal vessels had led to hemorhagic infarction of the retina and subsequent neovascular glaucoma. Zamir *et al.* [14] described a-2-year-old child with juvenile xanthogranulomatous disorder presenting as an unilateral chronic refractory uveitis without any skin or systemic findings with a painful blind eye and the diagnosis of juvenile xanthogranulomatous disease could be established following the histological, immunhistochemical and electron microscopic studies of the enucleated eye. Both anterior and posterior segments were involved including the retina and subretinal space.

In our case, microscopic examination revealed diffuse infiltrate of histiocytes with foamy or granular cytoplasm including only a few Touton giant cells extending towards the lamina propria of the conjunctiva. A few neutrophils, lymphocytes and plasma cells were also seen within the histiocytic infiltrate. As there was no necrosis and fibrosclerosis, we excluded the diagnosis of necrobiotic xanthogranuloma and Erdheim-Chester disease, respectively. Additionally, in our case, the histiocytes were positive for CD68 whereas negative for CD1a and S100. With these immunohistochemical staining pattern we excluded the Langerhans cell histiocytosis-histiocytosis X spectrum.

In light of our literature search, we believe that our case is the only adult case characterized with posterior segment manifestations of the adult xanthogranulomatous eye disease in association with the anterior segment involvement and we believe the present case represents a Class 2 type histiocytic disorder.
